# CsPbCl_3_‐Driven Low‐Trap‐Density Perovskite Grain Growth for >20% Solar Cell Efficiency

**DOI:** 10.1002/advs.201800474

**Published:** 2018-05-16

**Authors:** Jiexuan Jiang, Zhiwen Jin, Fei Gao, Jie Sun, Qian Wang, Shengzhong (Frank) Liu

**Affiliations:** ^1^ Key Laboratory of Applied Surface and Colloid Chemistry Ministry of Education Shaanxi Key Laboratory for Advanced Energy Devices Shaanxi Engineering Lab for Advanced Energy Technology School of Materials Science & Engineering Shaanxi Normal University Xi'an 710119 P. R. China; ^2^ Dalian National Laboratory for Clean Energy iChEM, Dalian Institute of Chemical Physics Chinese Academy of Sciences Dalian 116023 P. R. China

**Keywords:** CsPbCl_3_, grain boundaries, perovskites, solar cells, trap density

## Abstract

Charge recombination in grain boundaries is a significant loss mechanism for perovskite (PVK) solar cells. Here, a new strategy is demonstrated to effectively passivate trap states at the grain boundaries. By introducing a thin layer of CsPbCl_3_ coating before the PVK deposition, a passivating layer of PbI_2_ is formed at the grain boundaries. It is found that at elevated temperature, Cl^−^ ions in the CsPbCl_3_ may migrate into the PVK via grain boundaries, reacting with MA^+^ to form volatile MACl and leaving a surface layer of PbI_2_ at the grain boundary. Further study confirms that there is indeed a small amount of PbI_2_ distributed throughout the grain boundaries, resulting in increased photoluminescence intensity, increased carrier lifetime, and decreased trap state density. It is also found that the process passivates only grain surfaces, with no observable effect on the morphology of the PVK thin film. Upon optimization, the obtained PVK‐film‐based solar cell delivers a high efficiency of 20.09% with reduced hysteresis and excellent stability.

Organic–inorganic lead halide perovskite (PVK), with its outstanding photophysical properties and versatility in low‐temperature solution processes,^1^, ^2^, ^3^, ^4^, ^5^, ^6^, ^7^, ^8^ is considered one of the most promising materials for thin‐film solar cells.^9^, ^10^, ^11^ In just the few years since its invention, the power conversion efficiency (PCE) of the organic–inorganic hybrid PVK solar cell (PSC) based on mixed cations and halides has been rapidly increased to as high as 22.7%.^12^, ^13^, ^14^

In improving the performance of PSCs, a series of strategies have been studied, such as interface material fabrication,^15^, ^16^, ^17^, ^18^ crystal‐growth engineering,^19^, ^20^, ^21^, ^22^, ^23^ optical engineering,^24^, ^25^, ^26^, ^27^ and composition engineering.^28^, ^29^, ^30^, ^31^, ^32^, ^33^ Significant achievements have been made in these areas. More recently, trap state passivation has also been found to be a key factor because the trap states in the PVK layer, especially in the grain boundaries,^34^, ^35^, ^36^, ^37^ can induce serious charge carrier recombination for reduced PSC performance. Unfortunately, these trap passivation methods usually trigger uncontrollable nucleation sites in the film, resulting in deteriorated PVK grain growth and crystallinity.^38^, ^39^, ^40^, ^41^ Hence, it is imperative to develop an effective method to passivate the grain boundary defects without affecting the crystallinity of the PVK film.

Herein, we demonstrate a new strategy to effectively passivate trap states at the grain boundaries. By introducing a thin layer of CsPbCl_3_ before the PVK deposition, a passivating layer of PbI_2_ is formed at the grain boundaries. It is found that the CsPbCl_3_ layer has no effect on the morphology of the PVK thin film beyond passivating the trap states at the grain boundaries. As a result, the compact PVK film has uniform morphology without pinholes, allowing us to fabricate high‐performance PSCs with efficiency of 20.09% and superior stability.

To study device performance, the planar structure (FTO/TiO_2_/PVK/spiro‐OMeTAD/Au) was employed using (FAPbI_3_)_0.85_(MAPbBr_3_)_0.15_ as an active PVK absorber material because of its optimized bandgap and phase stability.^42^ For the material preparation, CsPbCl_3_ quantum dots (QDs) were sandwiched between TiO_2_ and PVK precursor layers. **Figure** **1**a schematically details the formation process of the CsPbCl_3_‐QDs‐driven low‐trap grain‐boundary FA_0.85_MA_0.15_PbI_2.55_ Br_0.45_ thin film. It is expected that the PVK thin film with high defect density was first produced by annealing the liquid FA_0.85_MA_0.15_PbI_2.55_Br_0.45_ precursor at 150 °C for 15 min. During the process, the Cl^−^ ions in the CsPbCl_3_ QDs diffuse into the PVK film via the grain boundaries, reacting with MA^+^ to form volatile MACl, which sublimes at the elevated temperature and leaves a surface layer of PbI_2_ that passivates the grain boundaries.^43^

**Figure 1:**
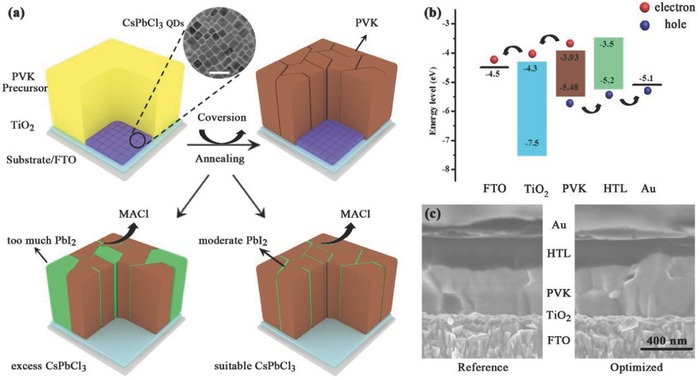
a) The conversion process for different CsPbCl_3_ contents. The inset is the TEM image of CsPbCl_3_ QDs, and the scale bar is 50 nm. b) Energy level diagram. c) The cross‐sectional SEM images of the devices based on the reference and CsPbCl_3_ optimized PVK film.

It should be noted that when a suitable amount of CsPbCl_3_ QDs is used to form just a surface layer of PbI_2_, the optimum passivation effect is observed. Any superfluous CsPbCl_3_ QDs will generate too much residual PbI_2_, thus inhibiting the charge transfer and collection.^44^ The energy level diagram of the PSCs is shown in Figure 1b, and the photogenerated carrier transport is illustrated as well.^45^, ^46^ The comparison of the cross‐sectional scanning electron microscopy (SEM) images (Figure 1c) of the devices with and without the CsPbCl_3_ QDs treatment indicates that the CsPbCl_3_ QDs have no obvious effect on the PVK grain growth beyond the surface passivation.

SEM, atomic force microscope (AFM), and energy‐dispersive X‐ray (EDX) are utilized to further investigate the composition and morphological evolution of the PVK films treated with different amounts of CsPbCl_3_ (0, 5, 20, and 50 mg mL^−1^). The SEM images of the representative PVK films are exhibited in **Figure** **2**a–d. All the obtained films display compact and uniform morphology without observable pinholes. Note that in the SEM images, components with different conductivities are displayed in different shades of gray.^47^, ^48^ The control film made without the CsPbCl_3_ QDs shows essentially all dark grains throughout the image, indicating only PVK grains without PbI_2_. When the CsPbCl_3_ QDs were introduced, white edges appeared around the PVK grain boundaries, indicating formation the PbI_2_ surface layers. The more CsPbCl_3_ QDs is used, the more PbI_2_ is observed. The white PbI_2_ phase covers almost half of the film when the CsPbCl_3_ concentration is increased to 50 mg mL^−1^. It is clear that the formation of PbI_2_ originated from the reaction of CsPbCl_3_ QDs with the PVK grain surfaces. Meanwhile, a similar phenomenon is also observed in the MAPbI_3_ film, as shown in Figures S1 and S2 and Table S1 in the Supporting Information.

**Figure 2:**
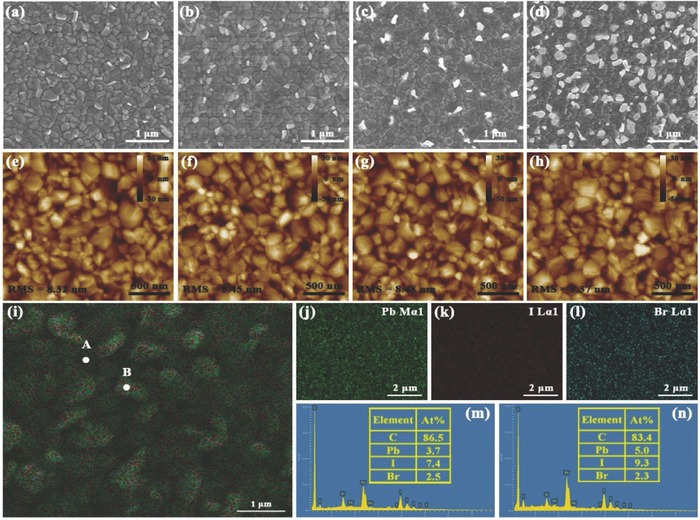
a–d) The SEM images and e–h) AFM images of PVK thin films (FA_0.85_MA_0.15_PbI_2.55_ Br_0.45_) formed using different amounts (0, 5, 20, and 50 mg mL^−1^, respectively) of CsPbCl_3_. i–l) The EDX elemental mappings and m,n) EDX spectra of PVK film using 50 mg mL^−1^ CsPbCl_3_ QDs at different locations.

However, the roughness and morphology of the films in the AFM images (Figure 2e–h) do not change with the addition of the various amounts of CsPbCl_3_. Such findings suggest that the CsPbCl_3_ only influences the component formation and has almost no effect on the morphology of the PVK thin film. The elemental mapping images and EDX analysis results are given in Figure 2i–n and confirm that the PVK films are composed of C, Pb, I, and Br but do not contain Cl. Figure 2m,n are, respectively, the EDX spectra of the dark (point A) and white (point B) grains in Figure 2i. The results demonstrate that residual PbI_2_ existed in the white grains, corresponding to the above SEM findings.

To further clarify the main role of CsPbCl_3_ in the formation of the PVK film, SEM images (Figure S3, Supporting Information) were acquired to observe the CsPbCl_3_ film before and after solvent treatment, which prove the CsPbCl_3_ film mainly adhered to the TiO_2_ film after PVK precursor treatment. **Figure** **3**a shows the X‐ray photoelectron spectroscopy (XPS) results of a TiO_2_ film deposited on FTO substrate (black line) and a CsPbCl_3_ QDs film treated with ethyl acetate (EA) and DMSO/GBL mixed solvent (3:7 v/v) on TiO_2_/FTO substrate. Obviously, O, Ti, and C are observed in both samples. In addition, the Cs 3d peaks, Pb 4f peaks, and Cl 2p peaks appear in the XPS spectrum of the CsPbCl_3_ QDs/TiO_2_/ FTO sample, which verifies the presence of CsPbCl_3_.

**Figure 3:**
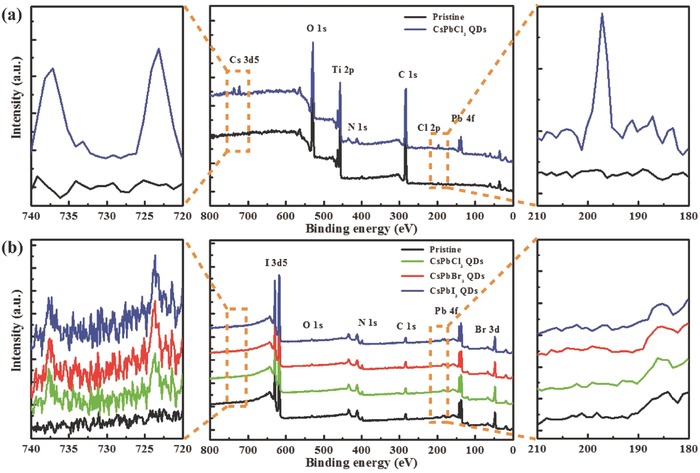
a) The XPS spectra of TiO_2_/FTO and CsPbCl_3_ QDs/TiO_2_/FTO film. b) The XPS spectra of pristine PVK thin films (FA_0.85_MA_0.15_PbI_2.55_ Br_0.45_) and films formed using 50 mg mL^−1^ of different components (CsPbCl_3_, CsPbBr_3_, and CsPbI_3_).

To understand the effect of Cl^−^ on the PVK films, XPS measurements were performed for CsPbX_3_‐driven (X = Cl, Br, I) PVK films. Compared with the pristine reference, the 3d peaks of Cs still exist for all the samples, whereas the 2p peaks of Cl disappear. Consistent with the speculation and findings of Figure 2, these results indirectly demonstrate the formation of volatile MACl. To further clarify whether Cs and Pb or Cl in the QDs play an important role in the passivation of PVK films, the performance of CsPbX_3_ (X = Cl, Br, I) QDs‐driven PVK‐based PSCs are illustrated and compared in Figures S4 and S5 and Table S2 in the Supporting Information. It is clear that CsPbBr_3_ and CsPbI_3_ have almost no effect on the evolution of the morphology of the PVK films. Also, they have negligible or negative impact on the device performance, suggesting Cl^−^ is critical for passivating the PVK films.

In order to study the effect of the CsPbCl_3_ QDs concentration on the structural and optical properties of PVK films, four representative samples with different contents of CsPbCl_3_ (0, 5, 20, and 50 mg mL^−1^) were selected as the research objects. To maintain identical test conditions, all the samples were prepared with the structure PVK/CsPbCl_3_/TiO_2_/FTO. The X‐ray diffraction (XRD) patterns (**Figure** **4**a) clearly confirm that the amount of PbI_2_ phase increases with increasing CsPbCl_3_, with is ascribed to the sublimation of MACl. These XRD results are coincident with those of the corresponding SEM images in Figure 2. The elemental proportions of Cs, Pb, Br, and I from the XPS measurements (Figure 4b) also indicate increasing PbI_2_ with increasing CsPbCl_3_, which is in agreement with the results of XRD and SEM.

**Figure 4:**
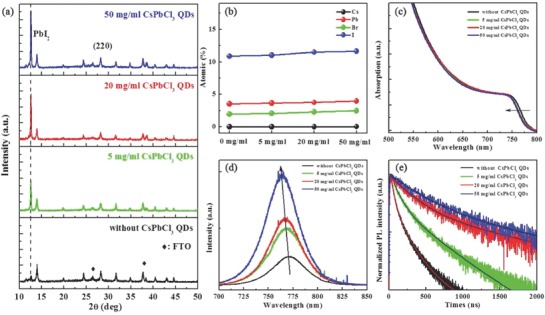
The structure and optical properties of PVK thin films (FA_0.85_MA_0.15_PbI_2.55_ Br_0.45_) formed using different amounts of CsPbCl_3_ (0, 5, 20, and 50 mg mL^−1^): a) XRD patterns, b) XPS elemental spectra, c) absorption spectra, d) PL spectra, and e) TRPL decay curves.

Figure 4c,d provides the absorption and photoluminescence (PL) spectra, respectively, of the pristine PVK films and those formed using different amounts of CsPbCl_3_. The observable bandgap widening and PL blueshift with increasing CsPbCl_3_ content mainly result from the existence of the PbI_2_ phase.^44^ It has been reported that the PbI_2_ could passivate the surface and grain boundaries of the PVK layer;^49^, ^50^ therefore, the PL intensity was remarkably increased with increasing CsPbCl_3_ content. Figure 4e compares the time‐resolved photoluminescence (TRPL) decay curves of the representative samples. The TRPL decay curves were fitted using a biexponential decay function [times (τ_i_) and amplitudes (*A*_i_)], with the relevant key parameters listed in Table S3 in the Supporting Information. The average recombination lifetime (τ_ave_) was estimated using^51^, ^52^, ^53^

(1) $$\tau_{\mathrm{ave}}=\frac{\sum Ai\tau_{i}^{2}}{\sum Ai\tau_{i}}$$

The lifetime is significantly prolonged from 0.13 to 1.24 µs with the increase of CsPbCl_3_ content, confirming that the PbI_2_ indeed passivates the defects in the grain boundaries in PVK films. The defects are considered recombination centers,^54^ and a longer lifetime is beneficial for extracting the photogenerated carriers.

Finally, the performances of different devices are compared to investigate the beneficial role of CsPbCl_3_ in the PSCs. **Figure** **5**a depicts the current density–voltage (*J*–*V*) curves of the devices fabricated without and with different CsPbCl_3_ contents. As the reference, the pristine device shows a PCE of 18.86%, with open‐circuit voltage (*V*_OC_), short‐circuit current density (*J*_SC_), and fill factor (FF) of 1.082 V, 22.87 mA cm^−2^, and 76.2%, respectively. With the introduction of the CsPbCl_3_, the device performance improved gradually with increasing QDs concentration. Even with the use of a small amount of CsPbCl_3_ QDs (5 mg mL^−1^), the device performance is improved significantly to a PCE of 19.35%, with the corresponding *V*_OC_, *J*_SC_, and FF improved to 1.101 V, 23.11 mA cm^−2^, and 76.1% respectively. When the CsPbCl_3_ content reached 20 mg mL^−1^, the PCE jumped to 20.09%. It is worth noting that both the *J*_SC_ and *V*_OC_ increased substantially. Given the prolonged lifetime, it is reasonable to attribute the improvement of the device performance to passivation of the traps by PbI_2_. However, further increasing the amount of CsPbCl_3_ QDs until there are too many CsPbCl_3_ QDs is not favorable to device performance, which is due to the wide bandgap (2.3 eV) of the resulting excess PbI_2_.^55^ The detailed parameters from the *J*–*V* curves are summarized in Table 2.

**Figure 5:**
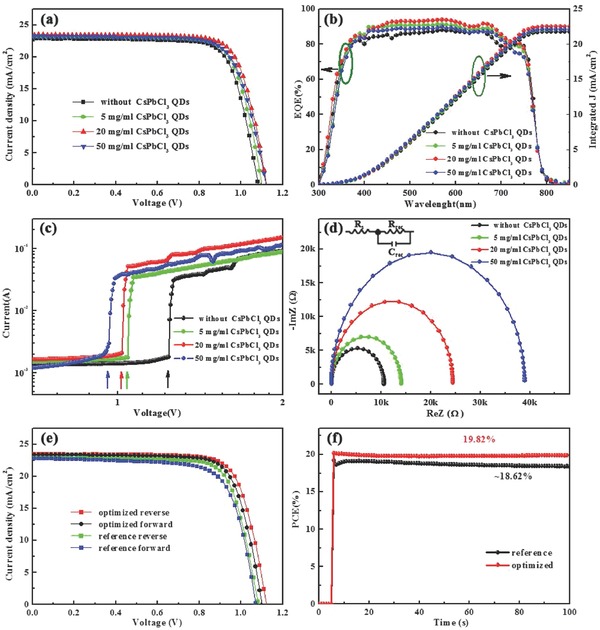
a) *J*–*V* curves. b) EQE spectra and integrated *J*_sc_. c) Dark *I*–*V* responses in capacitor‐like devices. d) Nyquist plots, with the inset presenting the equivalent circuit. e) *J*–*V* curves from both reverse scans and forward scans. f) PCE stability.

The typical external quantum efficiency (EQE) spectra of the devices are shown in Figure 5b. In the visible region, the EQE is highest for the device formed using 20 mg mL^−1^ CsPbCl_3_ QDs. The results further indicate that the CsPbCl_3_ QDs can enhance the device performance. It should be noted that the *J*_SC_ values calculated using the EQE curves are consistent with those from the *J*–*V* measurements (errors less than 5%), indicating that the latter is well calibrated. The trap state densities (*n*_t_) in all the representative devices were estimated from the dark current–voltage (*I*–*V*) curves (Figure 5c). The applied voltage at the kink point, where the current increases and becomes nonlinear, is defined as the trap‐filled limit voltage (*V*_TFL_), which is determined by the defect density. The following equation relates *V*_TFL_ to *n*_t_ 56

(2) $$V_{\mathrm{TFL}}=\frac{en_{t}L^{2}}{2\varepsilon \varepsilon_{0}},$$

where *e* is the elementary charge, *L* is the film thickness, ε is the relative dielectric constant, and *ε_0_* is the vacuum permittivity. The calculated values of *n*_t_ are shown in **Table** **1** for devices based on the pristine PVK films and those based on PVK films formed using different CsPbCl_3_ QDs contents. Distinctly, the trap state density decreases with increasing CsPbCl_3_ QDs amount. The reduced trap state density demonstrates the considerable improvement of the PVK film quality, resulting in the enhancement of the device performance. In parallel, the reduced trap state density is consistent with the prolonged lifetime in Figure 4e.

**Table 1:** Performance comparison of the PSCs based on different PVK films (extracted from Figures 4 and 5)

CsPbCl_3_ [mg mL^−1^]	*J*_SC_ [mAcm^−2^]	*V*_OC_ [V]	FF [%]	PCE [%]	*J*_SC(EQE)_ [mAcm^−2^]	τ_ave_ [µs]	VTFL [V]	*n*_t_ [cm^−3^]	τ_n_ [µs]
0	22.87	1.082	76.2	18.86	21.76	0.13	1.24	1.37 × 10^17^	45.3
5	23.11	1.101	76.1	19.35	22.11	0.31	1.05	1.16 × 10^17^	71.2
20	23.45	1.122	76.4	20.09	22.55	1.07	1.02	1.13 × 10^17^	140.2
50	23.27	1.121	76.6	19.46	22.13	1.24	0.97	1.07 × 10^17^	276.5

To further elucidate the role of CsPbCl_3_ on the improved performance of PSCs, the carrier transport process and recombination behaviors are investigated by electronic impedance spectroscopy (EIS).^57^, ^58^ The Nyquist plots for the devices without and with different CsPbCl_3_ concentrations measured in the dark are shown in Figure 5d. The equivalent circuit model comprised of the series resistance (*R*_s_), the recombination resistance (*R*_rec_), and capacitance (*C*_rec_) is depicted in the inset of Figure 5d. In the high‐frequency region, the *X*‐axis intercept is the equivalent *R*_s_. The main arc is associated with the recombination process. The EIS parameters from the Nyquist plots are summarized in Table S4 in the Supporting Information. Clearly, the value of *R*_s_ is decreased from 11.2 to 9.6 Ω after the CsPbCl_3_ modification. The reduced *R*_s_ indicates that the introduction of the CsPbCl_3_ QDs facilitates the carrier transport. However, when the concentration of CsPbCl_3_ QDs is 50 mg mL^−1^, the *R*_s_ is increased to 11.7 Ω, which is due to the excess PbI_2_ induced by the CsPbCl_3_. For *R*_rec_, the increasing values with increasing amount of CsPbCl_3_ mean that the carrier recombination is reduced. Given the identical conditions for all the PSCs, the increased *R*_rec_ could be mainly ascribed to the microstructure evolution resulting from the effect of the CsPbCl_3_ addition. Meanwhile, from the complex plane curves, the effective lifetimes (τ_n_) were extracted as the reciprocals of the frequencies corresponding to the peaks of the semicircles in the Cole–Cole plots.^59^ The τ_n_ for the optimized device (140.2 µs) is considerably longer than that of the pure PVK (45.3 µs), indicating that photogenerated carriers have more time to transport to the anode before possible recombination.

Hysteretic is a notorious behavior for planar PSCs. Figure 5e shows typical *J*–*V* curves of the devices measured using both reverse and forward scan directions, with key device parameters listed in **Table** **2**. It should be noted that *J*–*V* hysteresis was substantially eliminated by the addition of CsPbCl_3_, while the reference cell showed obvious hysteresis. The hysteresis index of the devices was defined by^60^

(3) $$\mathrm{Hysteresis}\;\mathrm{index}=\frac{{\mathrm{PCE}}_{\mathrm{reverse}}-{\mathrm{PCE}}_{\mathrm{forward}}}{{\mathrm{PCE}}_{\mathrm{reverse}}}$$

**Table 2:** Static figures of merit for the reference and CsPbCl_3_ QDs optimized devices

Device	Scanning mode	*J*_SC_ [mAcm^−2^]	*V*_OC_ [V]	FF [%]	PCE [%]	Hysteresis index
Reference	reverse	22.87	1.082	76.2	18.86	0.055
	forward	22.76	1.071	73.1	17.83	
Optimized	reverse	22.45	1.122	76.4	20.09	0.027
	forward	22.34	1.100	76.2	19.55	

Clearly, the hysteresis index for the optimized device is 0.027, lower than 0.055 for the reference formed without CsPbCl_3_ addition.

To ensure the *J*–*V* measurement is reliable, the PCEs of the devices were recorded as a function of time with the cells biased at their respective *V*_MP_ (voltage at the maximum power point, in this case 0.88 V for the reference and 0.90 V for the optimized device), as shown in Figure 5f. Clearly, the optimized device shows stable performance, while for the reference one, it exists some fluctuations which should be caused by the process of filling the uncertainty traps among grain boundaries under illumination. In general, both the stabilized PCE values are very close to those obtained by the direct *J*–*V* measurements. To confirm the process reproducibility, 50 individual devices (each) were fabricated from the reference and optimized films using the same procedure, with the statistical distribution of the key *J*–*V* parameters (*V*_OC_, *J*_SC_, FF, and PCE) presented in Figure S6 in the Supporting Information. All the key parameters exhibited fairly narrow distributions, indicating that the process was reproducible.

In summary, we adopted a thin CsPbCl_3_ QDs film to finely control the PbI_2_ content in planar PSCs, and we found that moderate residual PbI_2_ could result in stable and high‐efficiency solar cells. By fine‐tuning the residual PbI_2_ in the PVK layer accordingly, we have achieved a stable and high PCE of 20.09% with reduced hysteresis. We believe that more efficient and stable planar PSCs can be obtained in the future by employing interface engineering, crystal‐growth engineering, optical engineering, and compositional engineering.

## Conflict of Interest

The authors declare no conflict of interest.

## Supporting information

Supplementary
