# Supplementary material for: CsPbCl3‐Driven Low‐Trap‐Density Perovskite Grain Growth for >20% Solar Cell Efficiency
Source: Adv Sci (Weinh). 2018 May 16;5(7):1800474. doi: 10.1002/advs.201800474 (PMC6051377; doi:10.1002/advs.201800474)
Supplement: Supplementary file 1 — Supplementary [file ADVS-5-1800474-s001.pdf]

Copyright WILEY-VCH Verlag GmbH & Co. KGaA, 69469 Weinheim, Germany, 2018.

ADVANCED SCIENCE
Open Access

Supporting Information

for *Adv. Sci.,* DOI: 10.1002/advs.201800474

$CsPbCl_3$-Driven Low-Trap-Density Perovskite Grain Growth for >20% Solar Cell Efficiency

Jiexuan Jiang, Zhiwen Jin, Fei Gao, Jie Sun, Qian Wang,* and Shengzhong (Frank) Liu**

Supporting Information

$CsPbCl_3$ Driven Low-Trap-Density Perovskite Grain Growth for >20% Solar Cell Efficiency

Jiexuan Jiang[1], Zhiwen Jin[1,*], Fei Gao[1], Jie, Sun[1], Qian Wang[1,*] and Shengzhong (Frank) Liu[1,2,*]

[1]Key Laboratory of Applied Surface and Colloid Chemistry, Ministry of Education; Shaanxi Key Laboratory for Advanced Energy Devices; Shaanxi Engineering Lab for Advanced Energy Technology; School of Materials Science & Engineering, Shaanxi Normal University, Xi'an, 710119, P. R. China.
E-mail: jinzhiwen@snnu.edu.cn, wangqian16@snnu.edu.cn

[2]Dalian National Laboratory for Clean Energy; iChEM, Dalian Institute of Chemical Physics, Chinese Academy of Sciences, Dalian, 116023, P. R. China
E-mail: szliu@dicp.ac.cn

Keywords: $CsPbCl_3$, grain boundary, perovskite, solar cells, trap density

Experimental Section

Materials Preparation

Synthesis of $CsPbX_3$ QDs: Cs_2CO_3 (0.8 g), 1-octadecene (ODE, 50 mL) and oleic acid (OA, 2mL) were added to a round bottom flask under vacuum for 30 min at 120 °C as Cs-oleate. PbX_2 (2 mmol), ODE (50 mL), OA (5mL) and oleylamine (OAm, 5mL) were stirred in another round bottom flask and degassed under vacuum at 120 °C for 1 hour before filling with N_2. Then, the Cs-oleate (8 mL) was swiftly injected into the reaction mixture at 170 °C and maintained at temperature for 5 sec. Then, the reaction was quenched by immediate immersion of the flask into an ice bath. Finally, the synthesized $CsPbX_3$ QDs were precipitated by adding 200 ml ethyl acetate (EA). This process was repeated twice and the $CsPbX_3$ QDs were dispersed in octane with different concentrations.

PVK precursor solution: $FA_{0.85}MA_{0.15}PbI_{2.55}Br_{0.45}$ solution (1.2 M) was prepared in a mixed solvent of DMF and DMSO with a volume ratio of 4:1(4:1 v/v). The molar ratios for PbI_2 /$PbBr_2$ and FAI/MABr were both fixed at 0.85:0.15, and the molar ratio

of (FAI+MABr)/(PbI_2 +$PbBr_2$) was fixed at 1:1. $MAPbI_3$ solution was prepared in a mixed solvent 1.2 M PbI_2/1.2 M MAI dissolved in a DMSO and Gamma-Butyrolactone (GBL) mixed solvent (3:7 v/v).

HTL solution preparation: A solution was prepared by dissolving Spiro-OMeTAD (90 mg), sulfonyl imide (Li-TFSI, 22 μL) solution (520 mg Li-TFSI in 1 mL acetonitrile) and tert-butylpyridine (TBP, 36 μL) in 1 mL of chlorobenzene.

All used solutions were filtered through a 0.4-μm pore PTFE filter and were stored in a dry nitrogen atmosphere.

Device Fabrication

Preparation of the TiO_2 blocking layer: Fluorine-doped tin oxide (FTO)-coated glass with a size of 25 × 25 mm^2 was washed sequentially with detergent, deionized water, acetone, and isopropanol with ultrasonication for 10 min each, and then were dried by N_2 and treated by an O_2 plasma. The clean substrate was immersed in a 40 mM $TiCl_4$ aqueous solution for 30 min at 70 °C and washed with distilled water and ethanol, followed by annealing at 200 °C for 30 min in air to form a compact n-type blocking layer of TiO_2.

Growth of the $CsPbX_3$ QDs film: The QDs layer was fabricated on the above-prepared TiO_2 layer at 2500 rpm by spin-coating the prepared QDs solutions with different concentrations. Immediately after the spin-coating, the prepared film was quickly dipped sequentially into saturated $Pb(OAc)_2$ EA solution and neat EA solution.

Growth of the PVK film: The prepared PVK precursor solution was spin-coated onto the QDs film at a speed of 4000 rpm for 20 s. CB (100 μL) was dropped onto the spinning substrate during the spin-coating step at 10 s before the end of the procedure. The film was then heated at 150 °C for 15 min.

Assembly of the PSCs: An HTL film was prepared by spin-coating the HTL solution onto the PVK film at 4000 rpm for 30 s. Finally, a gold electrode with a thickness of ~70 nm was thermally evaporated onto the Spiro-OMeTAD-coated film to finish the device fabrication.

The bare devices without any encapsulation were stored and tested upon exposure to the ambient environment (in air at relative humidity of ~30% at 25 °C).

Characterization

The film surface morphology and cross-sections were characterized by SEM and EDS (Jeol SU-8020). The TEM images were obtained using an FEI Tecnai T20 equipped with a Gatan SC200 CCD camera and LaB6 filament operated at 200 kV. The XPS measurements were performed in a VG ESCALAB MK2 system with monochromatized Al Kα radiation. XRD patterns were performed on a DX-2700 with Cu Kα radiation (λ = 0.15418 nm). Absorbance spectra were collected using a Shimadzu UV-3600 double beam spectrometer. PL spectra were measured using a

Horiba Jobin Yvon Fluoro Log2 spectrofluorometer. TRPL spectra were acquired according to a time-correlated single photon counting method using an Edinburgh Instruments FLS920 fluorescence spectrometer. The laser diode is capable of a repetition rate of 80 MHz; however, the repetition rates were adjusted as appropriate to observe the full decay. J-V curves were measured at 25 °C under AM1.5G (100 mW/cm^2) illumination (scan rate: 0.5 V/s, both forward (from I_{SC} to V_{OC}) and reverse (from V_{OC} to I_{SC}) scan modes). Here, the bare devices without any encapsulation were stored and tested upon exposure to the ambient environment (in air at relative humidity of 25%~35% at 25 °C). A black cardboard mask with a window area of 0.09 cm^2 was clipped onto the glass side to define the active area of the cell. The spectral response was taken by an EQE measurement system (QEX10, PV Measurement), which was equipped with a monochromator, a lock-in amplifier, a Xe lamp, and a current-voltage amplifier. Prior to the use of the light, the spectral response and the light intensity were calibrated using a mono-silicon detector. The frequency-dependent capacitances (C-f) of the devices were obtained by a semiconductor device analyzer (Agilent Technologies, B1500A). An AC voltage perturbation of 20 mV and a constant bias at the open voltage was maintained. Each spectrum was measured covering the range from 1 KHz to 1 MHz.

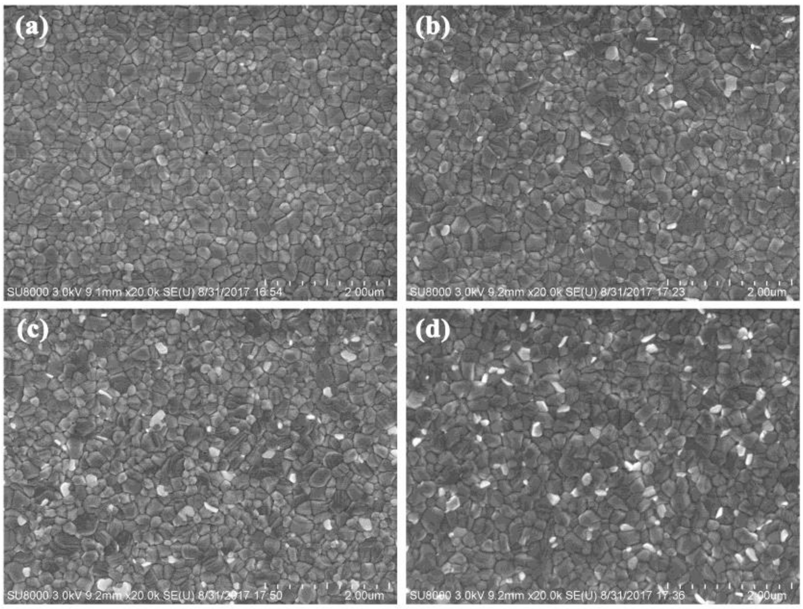

Figure S1. (a)-(d) The SEM images of perovskite thin films ($MAPbI_3$) formed using different amounts (0, 5, 20 and 50 mg/ml, respectively) of $CsPbCl_3$.

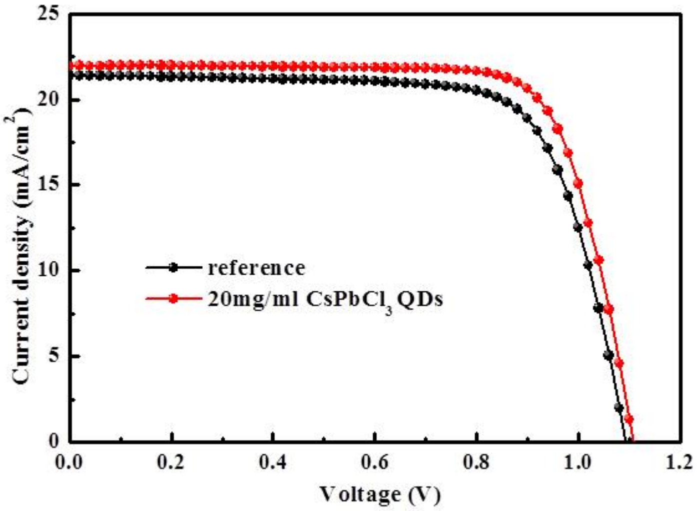

Figure S2. Comparison of the *J*-*V* characteristics of the reference and 20 mg/ml $CsPbCl_3$ QDs optimized perovskite ($MAPbI_3$) devices.

Table S1. Device performance parameters extracted from **Figure S2**.

$MAPbI_3$	J_{SC} ($mAcm^{-2}$)	V_{OC} (V)	FF (%)	PCE (%)
Reference	21.37	1.091	73.3	17.10
Optimized	21.94	1.108	76.3	18.56

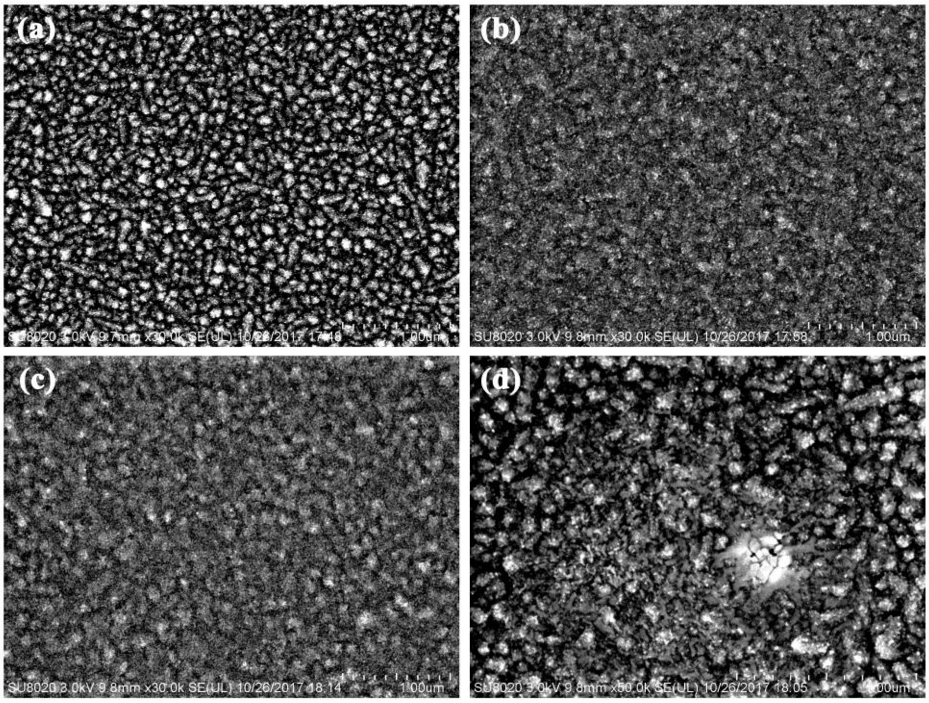

Figure S3. SEM images of (a) FTO, (b) $CsPbCl_3$ QDs film, (c) EA-treated $CsPbCl_3$ QDs film, and (d) $CsPbCl_3$ QDs film treated with EA and DMSO/GBL mixed solvent (3:7 v/v).

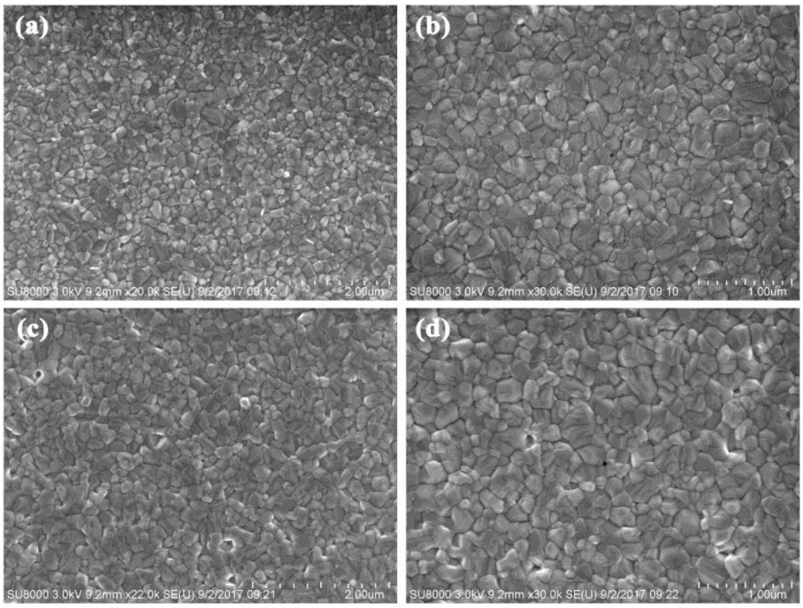

Figure S4. SEM images for 50 mg/ml QDs-driven perovskite thin films: (a) and (b) for $CsPbBr_3$, (c) and (d) for $CsPbI_3$.

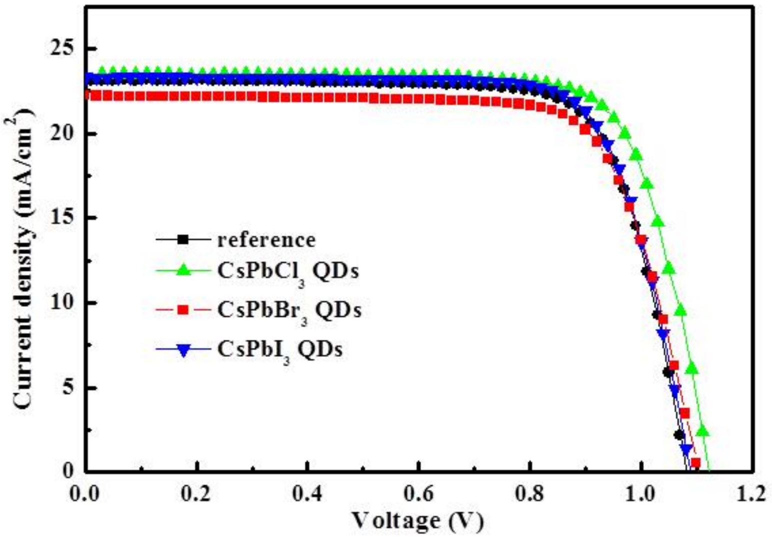

Figure S5. Comparison of the *J-V* characteristics of devices based on perovskite thin films ($FA_{0.85}MA_{0.15}PbI_{2.55}$ $Br_{0.45}$) driven with different QDs ($CsPbCl_3$, $CsPbBr_3$ and $CsPbI_3$).

Table S2. Device performance parameters extracted from **Figure S5**.

Device	J_{SC} (mAcm^{-2})	V_{OC} (V)	FF (%)	PCE (%)
reference	22.87	1.082	76.2	18.86
$CsPbCl_3$ QDs	23.45	1.122	76.4	20.09
$CsPbBr_3$ QDs	22.22	1.103	74.4	18.24
$CsPbI_3$ QDs	23.30	1.087	76.0	19.25

Table S3. TRPL parameters extracted from **Figure 4e**.

$CsPbCl_3$ (mg/ml)	τ_{ave} (μs)	τ_1 (μs)	τ_2 (μs)	% of τ_1	% of τ_2
0	0.13	0.22	0.046	16.1	83.9
5	0.31	0.44	0.097	26.8	73.2
20	1.07	1.5	0.27	24.8	75.2
50	1.24	1.72	0.37	28.2	71.8

Table S4. EIS parameters extracted from **Figure 5d**.

$CsPbCl_3$ (mg/ml)	R_s (Ω)	R_{rec} (kΩ)	C_{tr} (nF)	τ_n (μs)	V_{TFL} (V)	n_t (cm^{-3})
0	11.2	10.6	4.27	45.3	1.24	1.37×10^{17}
5	9.8	14.8	5.99	71.2	1.05	1.16×10^{17}
20	9.6	24.5	5.16	140.2	1.02	1.13×10^{17}
50	11.7	38.9	5.49	276.5	0.97	1.07×10^{17}

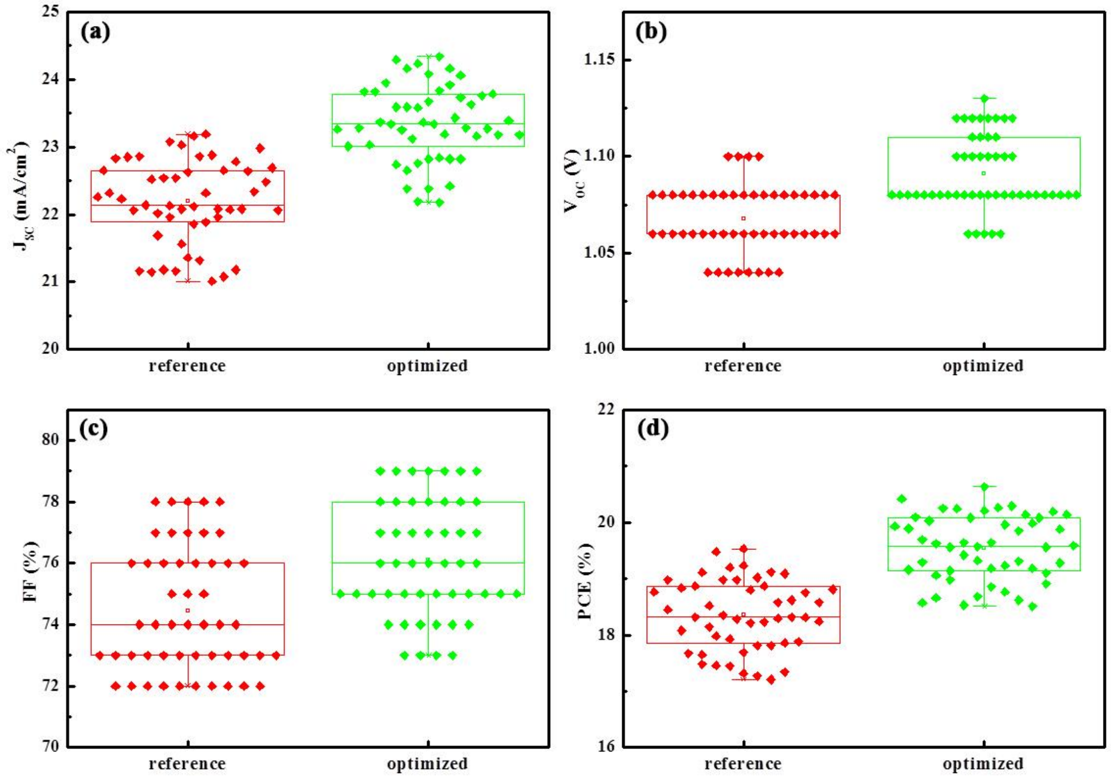

Figure S6. 50 individual devices of each type (pristine and optimized) based on $FA_{0.85}MA_{0.15}PbI_{2.55}$ $Br_{0.45}$ films were fabricated: (a) J_{SC} distributions; (b) V_{OC} distributions; (c) FF distributions; and (d) PCE distributions.
